# A Cross-Sectional Examination of Physical Activity Levels and Their Socio-Demographic Determinants in Southern Tanzania

**DOI:** 10.3390/ijerph15061054

**Published:** 2018-05-23

**Authors:** Beverly Msambichaka, Ramadhani Abdul, Salim Abdulla, Paul Klatser, Marcel Tanner, Ramaiya Kaushik, Bettina Bringolf-Isler, Eveline Geubbels, Ikenna C. Eze

**Affiliations:** 1 Ifakara Health Institute, Kiko Avenue, Dar es Salaam P.O. Box 78373, Tanzania; msambichakabeverly@gmail.com (B.M.); rabdul@ihi.or.tz (R.A.); sabdulla@ihi.or.tz (S.A.); egeubbels@ihi.or.tz (E.G.); 2 Swiss Tropical and Public Health Institute, Socinstrasse 57, CH-4002 Basel, Switzerland; marcel.tanner@swisstph.ch (M.T.); bettina.bringolf@swisstph.ch (B.B.-I.); 3 University of Basel, Petersplatz 1, CH-4003 Basel, Switzerland; 4 Athena Institute, Vrije Universiteit/Free University, 1081 HV Amsterdam, The Netherlands; paulklatser@gmail.com; 5 Shree Hindu Mandal Hospital, Chusi St, Dar es Salaam P.O. Box 581, Tanzania; ceo@hindumandal.org

**Keywords:** physical activity, vigorous physical activity, determinants, leisure, Tanzania, cross-sectional study, adults

## Abstract

Physical activity is essential for healthy aging. Evidence suggests that vigorous-intensity physical activity (VPA) may be more beneficial than moderate-intensity physical activity (MPA). We examined physical activity levels (MPA, VPA and total physical activity), and their socio-demographic determinants in 2311 participants (15–93 years; 68% women) of the MZIMA Open Community Cohort, who had complete relevant data. Physical activity levels were estimated in minutes per week across three domains—work, leisure and transport. We created three outcome variables: low MPA (<150 min per week of MPA), low VPA (<75 min per week of VPA) and insufficient physical activity (IPA: <150 min per week of total physical activity) and applied sample-weighted multivariable logistic regression to assess associations with potential socio-demographic determinants. Prevalence of IPA, low MPA and low VPA were 25%, 26% and 65% respectively. IPA and low MPA were correlated (Spearman R = 0.98; *p* < 0.001). Work, leisure and transport contributed 54%, 25% and 21% to total physical activity respectively. IPA and low VPA were significantly associated with female sex, lower education, non-manual occupation and frequent fruit consumption. We observed significant differences by sex (*P_heterogeneity_* < 0.001), on the associations between education and IPA, and between age, occupation and low VPA. In conclusion, low levels of VPA, which were more pronounced in women, support the monitoring and promotion of VPA alongside overall physical activity. Leisure-related activities should also be promoted towards gains in vigorous-intensity and total physical activity in this setting.

## 1. Introduction

Physical activity is a key factor in the prevention of non-communicable diseases (NCD) [1]. In order to maximize its health benefits, more physical activity should be desired, while avoiding injury or harm [2]. A 75 min per week of vigorous-intensity physical activity (VPA) or 150 min per week of moderate-intensity physical activity (MPA) or their combination (150 min per week of total physical activity calculated as MPA+(VPA*2)) is currently recommended to reduce the risk of major NCDs [3,4]. Yet, levels of physical activity around the world fall below these recommendations, contributing to 3.2 million mortalities and 13.4 disability-adjusted life years due to cardiovascular diseases, diabetes, and cancers [5,6]. Several studies showed that VPA was associated with better physical function and cardiovascular health compared to moderate activity [2,7,8,9,10]. Hence, VPA should be encouraged and described alongside total physical activity to better capture the state of physical activity towards targeted interventions, in any population.

Many low- and middle-income countries (LMICs) like Tanzania display sufficient physical activity mainly via their occupations and transport [11,12,13]. Manual occupations such as farming and transport via foot or bicycle therefore help to meet the World Health Organization (WHO) recommendation for physical activity. The development of machines has made occupations less strenuous, leading to less VPA [5,14]. According to survey results from Uganda, 94.3% met the WHO recommendation for physical activity, but had low VPA and high sedentary behaviour [13]. A study in Mozambique also showed predominance of MPA than VPA [15]. Therefore, a concurrent study of VPA and total physical activity levels is warranted. This will help in the identification of areas for intervention, and with longitudinal evidence, an early identification of transition into inactivity could be achieved and addressed, towards attainment and maintenance of optimal overall physical activity levels.

Various socio-demographic factors may influence physical activity levels. For instance, occupations are a major contributor to physical activity, especially those which are strenuous in nature [16,17]. Gender disparity in physical activity is commonly reported, with higher levels in men than women [12,18]. Differences in responsibilities at the household level and in choice of occupation may influence this disparity [17,19,20,21], which is also evident in younger populations [22,23]. The mutual influence between NCD risk factors, where for instance smokers may tend to engage in more physical activity to compensate for the adverse effect of smoking, has been of interest [24,25,26,27], but has not been widely studied in African populations. 

Given the on-going epidemiologic transition, evidence on determinants of NCD risk factors including physical activity in LMICs, is essential for the development of population-specific public health interventions. This paper therefore explores the distribution of physical activity levels and their socio-demographic and lifestyle determinants, with focus on gender differences, in a Tanzanian population.

## 2. Materials and Methods

### 2.1. Study Design and Population

This was a cross sectional study using data from the second survey of the MZIMA Open Community Cohort which has been described elsewhere [28]. The cohort is lodged in the Ifakara Urban Health and Demographic Surveillance System [29]. This MZIMA survey occurred between May 2014 and September 2015 and included 4274 participants aged at least 15 years who were recruited from Mlabani and Viwanja Sitini areas of Ifakara town. Among other health indicators, the survey collected information on NCDs, socio-demographic and lifestyle characteristics including physical activity. The present study included only participants of the MZIMA cohort who responded to all physical activity questions and had all relevant socio-demographic and lifestyle variables for analyses.

Ethics approvals for the MZIMA Open Community Cohort were obtained from the Ifakara Health Institute Institutional Review Board (IHI/IRB/AM/01-2014) and the National Institute for Medical Research (NIMR/HQ/R.8a1Vol.IX/I320). All participants provided informed written consent to participate in the study. 

### 2.2. Measurement of Physical Activity 

Questions exploring domains of physical activity were based on the WHO Stepwise approach for surveillance tool for NCDs and their risk factors (STEPS) in member countries [30]. The physical activity questions, which have been validated in different settings [31,32,33], assessed MPA and VPA from work, transport (by foot or bicycle) as well as leisure-related activities. The merits of this questionnaire include the assessment of different physical activity domains, provision of activity examples to improve the understanding of interviewees, and the limited need to define leisure time for retirees and older adults [34]. The study questions used to assess physical activity are shown in Table 1. Data collection was done electronically using tablets in Open Data Kit format [35] and automatic skip patterns (the skipping of questions when they are not relevant based on a preceding response) were applied to minimize faulty entries.

Based on the participants’ responses, we calculated the following: (i) VPA as the sum of the product of time spent on vigorous-intensity activity and number of days in a week, across domains of physical activity (work, transport or leisure-related); (ii) MPA was evaluated as the sum of the product of time spent on moderate-intensity activity and number of days in a week, across domains of physical activity (work, transport or leisure-related) and (iii) total physical activity as the sum of (VPA*2) and MPA in min per week [4]. Based on the WHO cut-offs, we defined three physical activity outcomes: (i) insufficient physical activity (IPA) as having less than 150 min per week of total physical activity (ii) low MPA as having less than 150 min per week of MPA and (iii) low VPA as having less than 75 min per week of VPA [4]. 

### 2.3. Measurement of Socio-Demographic and Lifestyle Indicators 

Participants also responded to socio-demographic and other lifestyle questions. Age was categorized into “below 25 years”, “25–below 50 years”, “50–below 60 years”, and “60 years and above”. Sex was categorized into “male” and “female”. Education categorize included “no formal education”, “primary education”, “secondary education” and “tertiary education”. Occupational status was dichotomized into “yes/no” representing having or not having an income-generating activity. Specific categories for those with occupations were as follows: “farming, fishing and livestock keeping”; “business owners”; “professionals (white collar jobs)”; “skilled manual workers (including drivers, carpenters, etc.)”; and “unskilled manual workers (including menial jobs)”. Religion was categorized into “Muslim”, “Catholic”, “Lutheran” and “others”. Ethnicity of participants was categorized into “Morogoro”, “Iringa”, “Shinyanga/Mwanza/Tabora”, “Kilimanjaro/Arusha”, “Ruvuma”, and “others”. Participants also responded to questions on their alcohol consumption (e.g., beers, wines, spirits or local brews) in the past 12 months, tobacco smoking (e.g., cigarettes, cigars or pipes) and frequency of consumption of fruits and vegetables in any form, in a usual week. Alcohol consumption was classified into “yes” or “no”, whereas smoking status was classified into “current smoker” or “never smoker/former smoker”. Frequency of fruit consumption and vegetable consumption in a usual week were classified as ≤3/>3 days per week respectively.

### 2.4. Data Analysis

We identified participants who provided complete responses to the physical activity questions, as well as having complete socio-demographic information. We summarized the characteristics of these participants included in the final sample: categorical variables were described as proportions whereas continuous variables, due to their non-normal distributions, were described as medians and interquartile ranges (IQR). We calculated the contributions of the major physical activity domains to the total physical activity, based on their mean values in the study sample. We assessed the prevalence of IPA, low MPA and low VPA, as well as their correlations using the Spearman correlation test. We tested socio-demographic differences in these physical activity outcomes using univariable logistic regression. In a further step, we performed mutually-adjusted logistic regression to test the independence of observed associations, as well as explore the potential influence of fruit, vegetable and alcohol consumption, and smoking as correlates or modifiers of physical activity. 

Due to the overrepresentation of women in the cohort (male to female ratio of 41%), we applied sex-specific sampling weights to the models that combined men and women. These weights were derived as the inverse of sex-specific ratios of our cohort and those of the general population in the study area (male to female ratio of 83%) obtained from the 2012 National Census [36]. Thus, sampling weights of 2.02 (83/41) and 1 were applied to men and women respectively. We also stratified the descriptive estimates and the regression models by sex for sex-specific estimates and differences in trends and associations. Since our study included only 54% of survey participants, we explored potential selection bias by comparing the socio-demographic characteristics between included and excluded participants. Finally, we performed sensitivity analyses by additionally applying the inverse of the probability of participation in the present analyses, derived from the overall study sample, to the sex-weighted combined univariable and multivariable models. 

All association results were expressed as Odds ratios (OR) and 95% confidence intervals (CI), and statistical tests were considered significant at alpha-value of 0.05. All analyses were performed using STATA version 14 (STATA Corporation, College Station, TX, USA). 

## 3. Results

A total of 2311 (54%) participants responded to questions in completeness and no ambiguity hence were included in the analysis. Age range of the participants was 15–93 years. Median (IQR) age of participants was 30 (19) years, with 15% being at least 50 years old. Women comprised two-thirds of our study sample (68%). More than three-quarters (76.6%) of this sample had formal education, with only 4.3% having tertiary education. The participants included mixed ethnic groups from all parts of the country, but the largest groups were from Morogoro region (58.5%), and farming was the main economic activity (Table 2). There were significant differences between men and women in relation to education and occupation, but there were no significant differences by age, religion and ethnicity (Table 2).

Compared to the included participants, the 1963 excluded participants were more likely to be female, younger, more educated, unemployed and non-smokers (Table 3). 

### 3.1. General Picture of Physical Activity in the Population

Majority of the population met the WHO recommendation for total physical activity, with an IPA prevalence of 25%. The respective prevalence of low MPA and low VPA was 26% and 65%. All three measures of physical activity were significantly different between men and women (*p* < 0.001), with men reporting higher physical activity at all levels than women (Table 2). Spearman correlation (R) between IPA and low MPA was 0.98 (*p* < 0.001) whereas the correlation between IPA and low VPA was 0.42 (*p* < 0.001). Hence, further regression models and results are presented for IPA and low VPA. 

Mean time spent on total physical activity was 2033 min per week. Mean min per week of domain-specific physical activity was 1089 (work), 434 (transport) and 510 (leisure). Thus, the contribution of work towards total physical activity was 54%, whereas the contributions of transport and leisure were 21% and 25% respectively. This trend was similar in men and women where the respective contributions of work, transport and leisure were 53%, 22% and 25% for men and 54%, 21% and 25% for women. Median (IQR) time spent on work, transport and leisure-related activities were 0 (1200), 360 (480) and 120 (600) min per week respectively and were all significantly higher in men than in women (*p* < 0.001). 

### 3.2. Association of Physical Activity with Socio-Demographic Factors

In the univariable models, factors associated with IPA and low VPA included female sex, having no education, less manual work, fruit intake, vegetable intake and no alcohol intake. Older age was associated with IPA but the association between age and low VPA was less apparent. Being a never- or former smoker was associated with low VPA, but not IPA. Ethnicity was neither associated with IPA nor low VPA (Table 4).

In the multivariable models, associations of IPA and low VPA with the socio-demographic factors generally remained, but were of lesser magnitude, and the associations with age, alcohol intake and smoking became non-significant (Table 4). Adjusted OR (95% CI) of IPA and low VPA for female sex were 1.42 (1.08–1.84) and 2.78 (2.26–3.43), respectively. Adjusted OR (95% CI) of IPA and low VPA for educational level (tertiary vs. no education) were 0.12 (0.06–0.22) and 0.20 (0.11–0.37) respectively, whereas adjusted OR (95% CI) of IPA and low VPA for occupation (farming vs. unemployed) were 0.67 (0.48–0.94) and 0.27 (0.20–0.36) respectively. Participants who reported frequent intake of fruits had higher odds of IPA (OR: 1.45; 95% CI: 1.21–1.74) and low VPA (OR: 1.57; 95% CI: 1.28–1.93) independent of other variables (Table 4).

### 3.3. Sex-Specific Association of Socio-Demographic and Lifestyle Factors with Physical Inactivity

For men, the determinants of IPA included educational level (OR for tertiary vs. no education: 0.05; 95% CI: 0.02–0.15) and fruit intake (OR: 1.82; 95% CI: 1.10–3.02) whereas the determinants of low VPA included age (OR for >50 years vs. <25 years: 2.10; 95% CI: 1.21–3.65), educational level (OR for tertiary vs. no education: 0.14; 95% CI: 0.06–0.31), occupation (OR for farming vs. unemployed: 0.40; 95% CI: 0.24–0.70) and smoking (OR: 0.56; 95% CI: 0.32–0.97). Ethnicity and alcohol intake were not significant determinants of either IPA or low VPA in men (Table 5). 

For women, the determinants of IPA included educational level (OR for tertiary vs. no education: 0.27; 95% CI: 0.13–0.57), occupation (OR for farming vs. unemployed: 0.64; 95% CI: 0.46–0.88) and fruit intake (OR: 1.35; 95% CI: 1.04–1.74) whereas the determinants of low VPA also included educational level (OR for secondary vs. no education: 0.25; 95% CI: 0.16–0.39), occupation (OR for farming vs. unemployed: 0.17; 95% CI: 0.12–0.23) and fruit intake (OR: 1.53; 95% CI: 1.20–1.97). Similar to our findings in men, ethnicity, and alcohol intake did not determine IPA or low VPA levels in the women and in contrast to our findings in men, age and smoking did not determine IPA or low VPA in women (Table 5).

Comparing the determinants of IPA between men and women showed significant differences in the association between educational level and IPA. The degree of protection conferred by formal education was stronger in men than women (*P_heterogeneity_* < 0.001). We also observed sex differences in the associations with low VPA. There were significant differences in the association with age (*P_heterogeneity_* < 0.001), educational level (*P_heterogeneity_* < 0.001) and occupation (*P_heterogeneity_* < 0.001). Although fruit intake was a significant determinant of IPA and low VPA for both men and women, the differences between men and women for both outcomes were non-significant. Association with alcohol consumption or smoking was also not significantly different between men and women, for both IPA and low VPA (Table 5).

The results of sensitivity analyses using the general models corrected for potential selection bias showed very consistent findings. The determinants of IPA and low VPA from the sensitivity models were the same as those from the models limited to those with complete data, in both univariable and multivariable regression models (Table 6).

## 4. Discussion

We found that although majority of the participants met the WHO recommendations for physical activity, VPA was very low and indicates the importance for promotion of VPA alongside MPA towards overall physical activity. Moreover, women were more inactive compared to men, independent of age, education, ethnicity and occupation. 

Approximately three-quarters (74%) of the study sample reported sufficient physical activity. This physical activity profile is comparable to the national average (83.3%) reported in the 2012 Tanzania STEPS survey. Similar to the 2012 survey, we observed higher levels of physical activity in men than women. Nevertheless, the actual proportion of active men (86.3%) and women (80.5%) were higher in the 2012 survey compared to the proportion of active men (81.4%) and women (72.1%) in our study. Furthermore, the daily average time spent on physical activity in general was also lower in our study compared to those reported in 2012 [11]. 

Low-income countries reportedly reduced their energy expenditure from work over a period of two decades [37]. Walking and cycling have also declined with the recent rise of informal modes of transport like motorized bike taxi. Although studies have not explicitly reported the decline of vigorous activities but have highlighted a growing prevalence of sedentary practices [23,26,38], a study from Mozambique reported that 75 min per week of VPA was uncommon [15]. Thus, there is need for a longitudinal investigation of physical activity trends especially in LMICs, for early identification of potential transitions to lower physical activity and inactivity. The finding of an inverse dose response between vigorous activity and mortality in a follow up study of more than 200,000 adults over 6.5 years supports the promotion of VPA for healthy aging [7]. In order to promote VPA through purposeful and leisure cycling and other forms of recreational activity in the LMIC context, there is need to establish a supporting environment [39,40,41] as well as awareness about its health benefits [42,43,44,45]. These may encourage the uptake of VPA in these settings.

Our findings showed that men were less vigorously active with increasing age, while women were more likely to be active with age. While our finding of somewhat higher activity with age agrees with some studies which reported older age to be associated with more MPA than VPA [46], other studies showed a general decline of physical activity with age, which was associated with major life events such as losing a spouse or retirement [47,48]. Improved understanding of the patterns of sex and age interactions as determinants of physical activity, will therefore contribute to interventions aimed at improving physical activity in old age.

In agreement with other studies [17,49,50], we found participants’ education and occupation to be significant determinants of their physical activity levels. Participants with higher educational attainment had lower risk of IPA. This could be attributed to increased awareness, acceptance and engagements in physical activity for health benefits. Interestingly, we observed a stronger protective effect of educational attainment on risk of IPA compared to VPA. This might also indicate higher awareness of overall physical activity than vigorous-intensity activity. Engagement in vigorous-intensity activity should be further promoted, by leveraging the already existing awareness of its health benefits. We also observed that farmers and manual workers had low risk of IPA, which was more apparent with VPA. This is not surprising given the strenuous nature of these occupations which provides avenues for physical activity. Although professionals and business owners were more educated than the farmers and manual workers, they had higher risk of IPA in comparison. In fact, the degree of protection among the professionals from being insufficiently active was non-significant in comparison to the unemployed. As these groups are more likely to lead sedentary lifestyles due to the nature of their occupation, reinforcements of the benefits of physical activity, as well as creating enabling environment in the work place for engagement will improve their uptake of physical activity. This is especially important given that median time spent on physical activity was zero minutes per week, despite work making the greatest contribution to overall physical activity. As demonstrated in a recent review [51], there is need for further exploration into modifiable factors that determine occupational physical activity especially in this and similar settings, towards improvement.

Of all the concurrent lifestyle factors investigated in this study, only fruit intake frequency showed a significant association with lower physical activity levels. Our finding of an inverse relationship between frequent fruit intake and physical activity may imply that participants, who are less active, tend to consume more fruits as compensation for their lower activity levels. As recently described [52] and supported by our findings with educational level, this reinforces an existing awareness of the health benefits of physical activity. However, we did not replicate this finding with other lifestyle factors and in contrast to our finding with fruit intake, another study reported higher healthy eating rates with higher levels of physical activity [53]. More studies are therefore needed to better understand these interactions between lifestyles as NCD risk factors, for effective public health interventions.

### Strengths and Limitations

This study describes in detail, the prevalent physical activity levels within the MZIMA cohort, and the influence of several socio-demographic and lifestyle factors. This study is a valuable contribution to evidence on the situation of physical activity in southern Tanzania. Our findings are consistent with models corrected for potential selection bias means that our findings may be generalized to the cohort. In addition, the distribution of some sociodemographic characteristics in the Morogoro region where the cohort is located is similar to an extent, to those of the study participants. Similar to the regional characteristics, men had higher literacy rates, higher employment rates in public and private sector, our sample comprised more Christians and the participants were mostly of Morogoro origin [28,36]. Thus, our findings may also be generalizable to an extent, to the region. We applied a novel approach by modelling the determinants of vigorous physical activity alongside the usually-reported total physical activity. 

Limitations of our study include its cross-sectional design, which limits our causal interpretation of the observed patterns. The physical activity questions were not specifically validated in our study setting but were already validated in similar settings to sufficiently capture physical activity. Recall bias may also have affected physical activity reports, but we expect this influence to be minimal due to the short recall period (7 days). Moreover, some important factors like awareness about importance of physical activity, access to physical activity facility including owning equipment such as a bicycle for travel could not be considered. We did not have information on sitting time or time spent doing household chores thus, we could not compute total metabolic equivalents which would better capture the overall physical activity level. The lack of information on household chores, which are predominantly undertaken by women in this study setting, could partly explain the observed sex differences in our study. Lastly, the sex-distribution of our sample might impact the generalizability of our results, but our models have accounted for oversampling, and the sex-specific findings remain valid for sex-specific inferences. Furthermore, there were no sex differences in age, religion or ethnicity distribution of our sample. Despite our correction for potential selection bias, some selection bias may still persist. More representative studies are therefore warranted to confirm these findings.

## 5. Conclusions

The majority of participants were within the WHO recommended levels of weekly physical activity. However, the low level of VPA calls for public health response with a priority towards women and white-collar workers. Given the rise of motorised transport in this and other LMIC settings undergoing transition, routine physical activity should be highly encouraged by improvements in recreational facilities in the community and workplace, and education for behavioural change. Advocacy for incorporation of physical activity into global and national public health agenda has been slow [14,54], and lessons learned from our study should enhance its implementation at the local level. Finally, more qualitative and quantitative research is needed to build evidence base and further understand the socio-demographic patterns of physical activity in LMICs.

## Figures and Tables

**Table 1:** Questions used to assess physical activity in the present study, based on the WHO STEPS survey questions.

Main Question	Follow on Questions
Does your work involve moderate- intensity activities that cause small increases in breathing or heart rate such as brisk walking, carrying light loads, fishing, herding animals for at least 10 min continuously?	In a typical week how many days do you do moderate-intensity work?
	How many minutes do you spend doing moderate-intensity work in one of those days
Does your work involve vigorous-intensity activities that causes large increases in breathing or heart rate like carrying or lifting heavy loads, digging or construction work, land tilling, harvesting, carpentry for at least 10 min continuously?	In a typical week how many days do you do vigorous-intensity work?
	How many minutes do you spend doing vigorous-intensity work in one of those days
Do you walk or use bicycle at least 10 min continuously to get to and from places?	In a typical week how many days do you do you walk or use bicycle at least 10 min continuously?
	How many minutes do you spend walking or using bicycle at least 10 min continuously in one of those days
Do you do moderate-intensity exercise, fitness or recreational activities that cause a small increase in breathing or heart rate such as brisk walking, cycling, swimming, netball, volleyball for at least 10 min continuously?	In a typical week how many days do you do moderate-intensity exercise?
	How many minutes do you spend doing moderate-intensity exercise in one of those days
Do you do vigorous-intensity exercise that causes large increases in breathing or heart rate like football, swimming, basketball, running for at least 10 min continuously?	In a typical week how many days do you do vigorous-intensity exercise?
	How many minutes do you spend doing vigorous-intensity exercise in one of those days

**Table 2:** Characteristics of study population included in the present study.

Variables	All ^a^	Males	Females	*P*-Value ^b^
	2311 (100%)	743 (100%)	1568 (100%)	
**Categorical Variables, N (%)**				**Chi-Squared Test**
Age groups				0.941
<25 years	882 (38.2)	282 (37.9)	600 (38.3)	
25–50 years	1080 (46.7)	346 (46.6)	734 (46.8)	
50 and above	349 (15.1)	115 (15.5)	234 (14.9)	
Educational level				<0.001
No Education	542 (23.5)	141 (19.0)	401 (25.6)	
Primary education	1119 (48.4)	328 (44.1)	791 (50.4)	
Secondary education	551 (23.8)	222 (29.9)	329 (21.0)	
Tertiary education	99 (4.3)	52 (7.0)	47 (3.0)	
Occupation				<0.001
Unemployed	687 (29.7)	171 (23.0)	516 (32.9)	
Business owners	309 (13.4)	76 (10.2)	233 (14.9)	
Professionals	89 (3.9)	45 (6.1)	44 (2.8)	
Skilled manual labour	144 (6.2)	67 (9.0)	77 (4.9)	
Unskilled manual labour	77 (3.3)	47 (6.3)	30 (1.9)	
Farming (including crop, livestock, fishing)	1005 (43.5)	337 (45.4)	668 (42.6)	
Religion				0.180
No religion	8 (0.3)	6 (0.8)	2 (0.1)	
Muslim	804 (34.8)	250 (33.6)	554 (35.3)	
Catholic	1239 (53.6)	404 (54.4)	835 (53.3)	
Lutheran	49 (2.1)	15 (2.0)	34 (2.2)	
Others	211 (9.1)	68 (9.2)	143 (9.1)	
Ethnicity				0.386
Morogoro region	1352 (58.5)	421 (56.7)	931 (59.4)	
Iringa region	282 (12.2)	86 (11.6)	196 (12.5)	
Shinyanga/Mwanza/Tabora regions	138 (6.0)	51 (6.9)	87 (5.6)	
Ruvuma region	349 (15.1)	116 (15.6)	233 (14.9)	
Other regions	190 (8.2)	69 (9.3)	121 (7.7)	
Consumption of fruits >3 days/week	1140 (49.3)	373 (50.2)	767 (48.9)	0.684
Consumption of vegetables >3 days/week	1680 (72.7)	484 (65.1)	1196 (76.3)	<0.001
Alcohol consumption in the past 12 months	333 (14.4)	163 (21.9)	170 (10.8)	<0.001
Current smoker	111 (4.8)	95 (12.8)	16 (1.0)	<0.001
Insufficient physical activity	576 (25)	138 (19)	438 (28)	<0.001
Low moderate physical activity	592 (26)	143 (19)	449 (29)	<0.001
Low vigorous physical activity	1498 (65)	350 (47)	1148 (73)	<0.001
Continuous variables, (Median (IQR))				Median test
Age in years	30 (19)	30 (21)	29 (18)	0.426
Minutes per week of moderate physical activity	720 (1560)	960 (1740)	540 (1350)	<0.001
Minutes per week of vigorous physical activity	0 (480)	180 (1080)	0 (150)	<0.001
Minutes per week of total physical activity	840 (2700)	1680 (3780)	660 (2040)	<0.001
Minutes per week of total physical activity due to work	0 (1200)	0 (2520)	0 (0)	<0.001
Minutes per week of total physical activity due to travel	360 (480)	420 (660)	240 (420)	<0.001
Minutes per week of total physical activity due to recreational activities	120 (600)	300 (840)	0 (420)	<0.001

Insufficient physical activity was defined as having less than 150 min per week of total physical activity (i.e., moderate physical activity + (2*vigorous physical activity)). Low moderate physical activity defined as having less than 150 min per week of moderate physical activity. Low vigorous physical activity defined as having less than 75 min per week of vigorous physical activity. ^a^ All proportions were adjusted for sex; ^b^ *p*-value of the difference between males and females for each variable.

**Table 3:** Summary of socio-demographic characteristics of the MZIMA cohort participants, stratified by inclusion status in the present study.

Categorical Variables (%)	Included	Excluded	Chi-Squared Test ^a^
	N = 2311	N = 1963	
Females	68	75	<0.001
Age groups			<0.001
<25 years	38.2	44.5	
25–50 years	46.7	40.8	
50 and above	15.1	14.7	
Educational level			<0.001
No education	23.5	11.0	
Primary education	48.4	51.5	
Secondary education	23.8	32.6	
Tertiary education	4.3	4.9	
Occupation			0.003
Unemployed	29.7	34.6	
Business owners	13.4	12.4	
Professionals	3.9	5.0	
Skilled manual labour	6.2	5.9	
Unskilled manual labour	3.3	3.2	
Farming (including crop, livestock, fishing)	43.5	38.9	
Religion			0.063
No religion	0.4	0.1	
Muslim	34.8	36.7	
Catholic	53.6	50.5	
Lutheran	2.1	2.9	
Others	9.1	9.8	
Ethnicity			0.003
Morogoro region	58.5	57.3	
Iringa region	12.2	10.3	
Shinyanga/Mwanza/Tabora regions	6.0	7.7	
Ruvuma region	15.1	14.0	
Other regions	8.2	10.7	
Consumption of fruits >3 days/week	49.3	49.1	0.859
Consumption of vegetables >3 days/week	72.3	72.1	0.682
Alcohol consumption in the past 12 months	14.4	13.5	0.393
Current smoker	4.8	3.6	0.056

^a^*p*-values of the difference between included and excluded participants, for each variable.

**Table 4:** Relationship between socio-demographic characteristics and physical activity.

Variable	Categories	Insufficient Physical Activity		Low Vigorous Physical Activity	
		Model 1	Model 2	Model 1	Model 2
		OR (95% CI)	OR (95% CI)	OR (95% CI)	OR (95% CI)
Sex	Men	Reference	Reference	Reference	Reference
	Women	1.70 (1.37–2.11) *	1.42 (1.09–1.84) *	3.06 (2.56–3.68) *	2.78 (2.26–3.43) *
Age group	<25	Reference	Reference	Reference	Reference
	25–50	0.79 (0.63–0.99) *	0.79 (0.58–1.08)	0.72 (0.59–0.88) *	1.01 (0.79–1.29)
	>50	1.51 (1.13–2.00) *	0.95 (0.63–1.44)	1.05 (0.79–1.39)	1.42 (0.99–2.01)
Education	No	Reference	Reference	Reference	Reference
	Primary	0.07 (0.05–0.09) *	0.06 (0.05–0.09) *	0.25 (0.19–0.33) *	0.19 (0.14–0.26) *
	Secondary	0.07 (0.05–0.10) *	0.06 (0.04–0.09) *	0.27 (0.20–0.37) *	0.17 (0.12–0.24) *
	Tertiary	0.13 (0.07–0.21) *	0.12 (0.06–0.22) *	0.30 (0.19–0.50) *	0.20 (0.11–0.37) *
Ethnicity	Morogoro	Reference	Reference	Reference	Reference
	Iringa	1.23 (0.90–1.67)	1.11 (0.79–1.58)	1.02 (0.76–1.35)	0.93 (0.68–1.27)
	Shinyanga/Mwanza/Tabora	1.47 (0.98–2.20)	1.40 (0.84–2.32)	0.97 (0.66–1.43)	0.98 (0.64–1.49)
	Ruvuma	1.07 (0.80–1.42)	0.94 (0.67–1.32)	0.85 (0.65–1.10)	0.82 (0.61–1.10)
	Others	1.01 (0.69–1.47)	0.98 (0.62–1.55)	1.05 (0.74–1.47)	0.99 (0.68–1.46)
Occupation	Unemployed	Reference	Reference	Reference	Reference
	Business owners	0.59 (0.41–0.81) *	0.90 (0.57–1.42)	0.77 (0.56–1.07)	0.85 (0.59–1.21)
	Professionals	0.40 (0.22–0.87) *	0.74 (0.35–1.56)	0.45 (0.27–0.73) *	0.62 (0.35–1.10)
	Skilled manual workers	0.33 (0.18–0.52) *	0.45 (0.23–0.90) *	0.44 (0.30–0.67) *	0.63 (0.42–0.94) *
	Unskilled manual workers	0.09 (0.03–0.34) *	0.07 (0.02–0.27) *	0.27 (0.16–0.45) *	0.34 (0.18–0.63) *
	Farming	1.10 (0.87–1.38)	0.67 (0.48–0.94) *	0.39 (0.31–0.49) *	0.27 (0.20–0.36) *
Fruit intake	≤3 days/week	Reference	Reference	Reference	Reference
	>3 days/week	1.48 (1.21–1.81) *	1.55 (1.22–1.96) *	1.45 (1.21–1.74) *	1.57 (1.28–1.93) *
Vegetable intake	≤3 days/week	Reference	Reference	Reference	Reference
	>3 days/week	1.24 (0.99–1.57)	1.06 (0.81–1.38)	1.25 (1.03–1.53) *	1.05 (0.84–1.32)
Alcohol intake	No	Reference	Reference	Reference	Reference
	Yes	0.51 (0.36, 0.70) *	0.72 (0.47, 1.10)	0.67 (0.52, 0.86) *	1.10 (0.81, 1.51)
Current smoker	No	Reference	Reference	Reference	Reference
	Yes	0.78 (0.48, 1.26)	0.95 (0.47, 1.93)	0.47 (0.32, 0.70) *	0.64 (0.40, 1.04)

Insufficient physical activity was defined as having less than 150 min per week of total physical activity (i.e., moderate physical activity + (2*vigorous physical activity)). Low vigorous physical activity defined as having less than 75 min per week of vigorous physical activity. Sampling weights were applied to all estimates to account for oversampling of females. * *p* < 0.05. Model 1: Univariable model; Model 2: Multivariable model including all variables presented in the table.

**Table 5:** Gender differences in the relationship between socio-demographic and lifestyle characteristics and physical activity.

Variable	Categories	Insufficient Physical Activity			Low Vigorous Physical Activity		
		Males	Females	*p*-value ^a^	Males	Females	*p*-value ^a^
		OR (95% CI)	OR (95% CI)		OR (95% CI)	OR (95% CI)	
Age group	<25	Reference	Reference	0.732	Reference	Reference	<0.001
	25–50	0.66 (0.35–1.25)	0.94 (0.69–1.29)		1.19 (0.78–1.84)	0.85 (0.63–1.15)	
	>50	0.91 (0.41–2.02)	1.18 (0.79–1.78)		2.10 (1.21–3.65) *	0.84 (0.55–1.29)	
Education	No	Reference	Reference	<0.001	Reference	Reference	0.001
	Primary	0.03 (0.02–0.06) *	0.10 (0.07–0.13) *		0.14 (0.09–0.23) *	0.23 (0.16–0.32) *	
	Secondary	0.02 (0.01–0.05) *	0.12 (0.08–0.18) *		0.12 (0.07–0.21) *	0.25 (0.16–0.39) *	
	Tertiary	0.05 (0.02–0.15) *	0.27 (0.13–0.57) *		0.14 (0.06–0.31) *	0.45 (0.17–1.16)	
Ethnicity	Morogoro	Reference	Reference	0.272	Reference	Reference	0.874
	Iringa	1.04 (0.49–2.23)	1.16 (0.79–1.71)		1.00 (0.61–1.66)	0.81 (0.55–1.18)	
	Shinyanga/Mwanza/Tabora	0.83 (0.32–2.17)	1.77 (1.05–3.01)		0.79 (0.41–1.53)	1.04 (0.59–1.82)	
	Ruvuma	0.70 (0.34–1.45)	1.07 (0.74–1.54)		0.87 (0.55–1.36)	0.82 (0.58–1.16)	
	Others	0.84 (0.35–1.99)	1.04 (0.65–1.68)		0.85 (0.48–1.48)	1.36 (0.82–1.26)	
Occupation	Unemployed	Reference	Reference	0.448	Reference	Reference	<0.001
	Business owners	1.06 (0.40–2.84)	0.80 (0.52–1.22)		1.33 (0.71–2.48)	0.52 (0.34–0.80) *	
	Professionals	0.69 (0.17–2.89)	0.79 (0.34–1.84)		0.68 (0.31–1.50)	0.53 (0.22–1.25)	
	Skilled manual workers	0.52 (0.17–1.60)	0.36 (0.16–0.80) *		0.53 (0.28–1.00) *	1.34 (0.63–2.86)	
	Unskilled manual workers	0.04 (0.004–0.32) *	0.12 (0.03–0.55) *		0.42 (0.20–0.88) *	0.28 (0.12–0.64) *	
	Farming	0.73 (0.36–1.51)	0.64 (0.46–0.88) *		0.40 (0.24–0.70) *	0.17 (0.12–0.23) *	
Fruit intake	≤3 days/week	Reference	Reference	0.382	Reference	Reference	0.804
	>3 days/week	1.82 (1.10–3.02) *	1.35 (1.04–1.74) *		1.62 (1.17–2.22) *	1.53 (1.20–1.97) *	
Vegetable intake	≤3 days/week	Reference	Reference	0.656	Reference	Reference	0.845
	>3 days/week	1.12 (0.66–1.90)	1.02 (0.75–1.39)		0.99 (0.71–1.38)	1.18 (0.88–1.57)	
Alcohol intake	No	Reference	Reference	0.391	Reference	Reference	0.471
	Yes	0.63 (0.30, 1.33)	0.83 (0.54, 1.29)		1.08 (0.71, 1.66)	1.00 (0.67, 1.47)	
Current smoker	No	Reference	Reference	0.141	Reference	Reference	0.959
	Yes	1.04 (0.44, 2.43)	0.45 (0.14, 1.47)		0.56 (0.32, 0.97) *	1.13 (0.26, 4.83)	

Insufficient physical activity was defined as having less than 150 min per week of total physical activity (i.e., moderate physical activity + (2*vigorous physical activity)). Low vigorous physical activity defined as having less than 75 min per week of vigorous physical activity. All estimates were from multivariable models including all variables presented in the table. ^a^ *p*-values of heterogeneity derived from multivariable models including interaction terms between sex and respective variables and applying sampling weights to account for oversampling of females. * *p*-value < 0.05.

**Table 6:** Relationship between socio-demographic characteristics and physical activity, corrected for potential selection bias.

Variable	Categories	Insufficient Physical Activity		Low Vigorous Physical Activity	
		Model 1	Model 2	Model 1	Model 2
		OR (95% CI)	OR (95% CI)	OR (95% CI)	OR (95% CI)
Sex	Men	Reference	Reference	Reference	Reference
	Women	1.68 (1.36–2.09) *	1.43 (1.10–1.84) *	3.13 (2.61–3.76) *	2.87 (2.33–3.54) *
Age group	<25	Reference	Reference	Reference	Reference
	25–50	0.81 (0.65–1.01)	0.80 (0.59–1.08)	0.71 (0.58–0.86) *	1.00 (0.79–1.27)
	>50	1.55 (1.18–2.05) *	0.95 (0.63–1.42)	1.04 (0.78–1.37)	1.38 (0.97–1.95)
Education	No	Reference	Reference	Reference	Reference
	Primary	0.08 (0.06–0.11) *	0.07 (0.05–0.09) *	0.26 (0.20–0.34) *	0.19 (0.14–0.26) *
	Secondary	0.10 (0.07–0.13) *	0.06 (0.04–0.09) *	0.28 (0.21–0.38) *	0.17 (0.12–0.24) *
	Tertiary	0.17 (0.10–0.28) *	0.12 (0.07–0.23) *	0.31 (0.19–0.51) *	0.20 (0.11–0.37) *
Ethnicity	Morogoro	Reference	Reference	Reference	Reference
	Iringa	1.22 (0.91–1.65)	1.12 (0.79–1.58)	1.01 (0.76–1.33)	0.92 (0.68–1.26)
	Shinyanga/Mwanza/Tabora	1.48 (1.00–2.20) *	1.41 (0.86–2.33)	0.98 (0.67–1.44)	0.98 (0.65–1.48)
	Ruvuma	1.07 (0.81–1.42)	0.95 (0.68–1.32)	0.84 (0.65–1.08)	0.82 (0.61–1.09)
	Others	0.99 (0.69–1.44)	0.97 (0.63–1.51)	1.05 (0.75–1.47)	0.99 (0.68–1.45)
Occupation	Unemployed	Reference	Reference	Reference	Reference
	Business owners	0.61 (0.43–0.87) *	0.91 (0.58–1.43)	0.76 (0.55–1.05) *	0.84 (0.59–1.20)
	Professionals	0.43 (0.23–0.80) *	0.76 (0.37–1.59)	0.45 (0.29–0.73) *	0.62 (0.35–1.10)
	Skilled manual workers	0.32 (0.18–0.56) *	0.44 (0.23–0.85) *	0.45 (0.30–0.68) *	0.64 (0.43–0.96) *
	Unskilled manual workers	0.09 (0.03–0.30) *	0.06 (0.02–0.25) *	0.27 (0.16–0.44) *	0.34 (0.19–0.62) *
	Farming	1.13 (0.90–1.41)	0.69 (0.50–0.95) *	0.38 (0.30–0.48) *	0.26 (0.20–0.35) *
Fruit intake	≤3 days/week	Reference	Reference	Reference	Reference
	>3 days/week	1.46 (1.20–1.78) *	1.52 (1.20–1.92) *	1.44 (1.21–1.73) *	1.57 (1.28–1.91) *
Vegetable intake	≤3 days/week	Reference	Reference	Reference	Reference
	>3 days/week	1.24 (0.99–1.55)	1.05 (0.80–1.37)	1.26 (1.04–1.53) *	1.05 (0.85–1.31)
Alcohol intake	No	Reference	Reference	Reference	Reference
	Yes	0.52 (0.38–0.72) *	0.74 (0.48–1.13)	0.67 (0.52–0.85) *	1.09 (0.80–1.49)
Current smoker	No	Reference	Reference	Reference	Reference
	Yes	0.80 (0.50–1.30)	0.96 (0.47–1.96)	0.47 (0.32–0.69) *	0.67 (0.42–1.08)

Insufficient physical activity was defined as having less than 150 min per week of total physical activity (i.e., moderate physical activity + (2*vigorous physical activity)). Low vigorous physical activity defined as having less than 75 min per week of vigorous physical activity. Sampling weights were applied to all estimates to account for oversampling of females. The inverse of the probability of participating in present analyses derived from base dataset, was also applied to all models. * *p*-value < 0.05. Model 1: Univariable model; Model 2: Multivariable model including all variables presented in the table.
